# High throughput clone screening on overexpressed hERG1 and Kv1.3 potassium channels using ion channel reader (ICR) label free technology

**DOI:** 10.1016/j.heliyon.2023.e20112

**Published:** 2023-09-19

**Authors:** Alberto Montalbano, Cesare Sala, Ginevra Chioccioli Altadonna, Andrea Becchetti, Annarosa Arcangeli

**Affiliations:** a Department of Experimental and Clinical Medicine, University of Florence, I-50134, Florence, Italy; b Department of Medical Biotechnologies, University of Siena, Strada delle Scotte, 53100, Siena, Italy; c University of Milano-Bicocca, Department of Biotechnology and Biosciences, Piazza della Scienza 2, I-20126, Milano, Italy

**Keywords:** Potassium channels, hERG1, hKv1.3, Rubidium efflux, ICR8000

## Abstract

Pharmacological studies aimed at the development of newly synthesized drugs directed against ion channels (as well as genetic studies of ion channel mutations) involve the development and use of transfected cells. However, the identification of the best clone, in terms of transfection efficiency, is often a time consuming procedure when performed through traditional methods such as manual patch-clamp. On the other hand, the use of other faster techniques, such as for example the IF, are not informative on the effective biological functionality of the transfected ion channel(s). In the present work, we used the high throughput automated ion channel reader (ICR) technology (ICR8000 Aurora Biomed Inc.) that combine atomic absorption spectroscopy with a patented microsampling process to accurately measure ion flux in cell-based screening assays. This technology indeed helped us to evaluate the transfection efficiency of hERG1 and hKv1.3 channels respectively on the HEK-293 and CHO cellular models. Moreover, as proof of the validity of this innovative method, we have corroborated these data with the functional characterization of the potassium currents carried out by the same clones through patch-clamp recordings. The results obtained in our study are promising and represent a valid methodological strategy to screen a large number of clones simultaneously and to pharmacologically evaluate their functionality within an extremely faster timeframe.

## 1 Introduction

The role of ion channels in modulating the key cellular functions of excitable cells has been repeatedly demonstrated for several decades. These observations have recently been updated with the increasing evidence of an important role mediated by these channels also in non-excitable cells. Among the multitude of members belonging to the ion channel families, voltage-gated potassium channels, and in particular hERG1 and Kv1.3, represent a class of ion channels of particular interest as their dysregulated functionality has been associated with several pathologies or even appears to be implicated in tumor development and progression [[1], [2], [3], [4], [5]].

The human *ether-á-go-go*–related gene (hERG1 or KCNH2) encodes the pore-forming subunit of the hERG1 (Kv11.1) potassium channel [6]. This channel is responsible for the rapidly activating delayed-rectifier current (*IKr*) which mediates the membrane repolarization of cardiomyocytes after a cardiac action potential and thereby prevents further arrhythmogenic premature depolarizations [7]. It has been shown that alterations in hERG1 function are responsible for the onset of long QT syndrome [[8], [9], [10], [11], [12]]. Moreover, this channel has been associated with different tumoral processes such as cell cycle progression, angiogenesis, invasiveness, metastasis formation and the channel results overexpressed in a variety of cancer cells [13,14]. It has also been shown its implication in gastric, pancreatic, breast cancer and esophageal squamous cell carcinoma, bladder cancer and osteosarcoma as well [[15], [16], [17]].

Regarding KCNA3 (Kv1.3), it has been demonstrated its critical role in thymocyte pre-clonal expansion [18] and its relevance for mediating the proliferation of different cancer lines [[19], [20], [21]] together with primary cells [[22], [23], [24]].

Unfortunately, although it is known that several types of Kv channels are linked to either altered proliferation or apoptosis induction, encouraging results have been obtained in cancer therapy in only a few cases. This is mainly due to that the available drug tools have non-tissue-specific and side effects that limit their therapeutic use. However recent studies show toxins, small molecules and specific antibodies as promising strategies [25].

As cellular model for the study of Kv channels we have chosen the Chinese Hamster Ovary (CHO) cell line, which is characterized by low endogenous inward and outward potassium currents, compared to other cell lines included HEK-293, which express members of the Kv1 and Kv3 families [26]. To increase the transfection efficiency and the number of stably selected clones, CHO cells have been transfected with linearized expression vectors using the electroporation methodology.

The traditional reference method for the study of ion channels is undoubtedly the patch-clamp technique. But, although it is able to provide extremely precise measurements and functional characterizations of undisputed scientific quality, this method is nevertheless particularly “slow” in measuring and processing data when it is used, in example, for the evaluation of the huge amount of compounds (i.e. isomers) that can be produced nowadays and then tested as ion channels modulators. In a comparable way, classical electrophysiological assays aimed at screening multiple cellular clones by evaluating the expression levels of an ion channel or, again, the screening of several different functional mutations introduced on an ion channel, represent other further examples where classical electrophysiological methods require particularly long execution times. In the present work we show how the use of the non-radioactive rubidium flux assay performed with the ICR8000 high-throughput automated ion channel and transporter reader that combine atomic absorption spectroscopy with a patented microsampling process (Aurora Biomed Inc.) represents a valid and faster alternative (or companion) in the evaluation of the expression levels of potassium ion channels (hERG1 and hKv1.3) in model cells, as well as their pharmacological-functional validation [[27], [28], [29], [30]]. However we observed a shift of the IC50 measured with Rb^+^ flux assay compared to those obtained with the patch clamp as already observed by other authors [29]. We attribute this discrepancy to the different drug delivery method (fast delivery puff with patch clamp vs. longer incubation needed for ICR8000 protocols). Furthermore, to complete the present study, we performed whole-cell patch-clamp recordings on the same cell lines used on ICR8000 as proof of the reliability of this innovative method.

## 2 Methods

**Cell maintenance**. Human Embryonic Kidney (HEK-293) cells which stably express the hERG1 potassium channel [31] were maintained at 37 °C in 5% CO2 in Dulbecco's Modified Essential Medium (Euroclone S. p.A., 20,016 Pero (MI)) containing the following: 10% fetal bovine serum (Euroclone S. p.A., 20,016 Pero (MI)), 10% l-glutamine (Euroclone S. p.A., 20,016 Pero (MI), and 800 μg/ml Geneticin (Gibco).

Chinese Hamster Ovary (CHO) cells which stably express the hKv1.3 potassium channel were maintained at 37 °C in 5% CO2 in HAM's F12 (Euroclone S. p.A., 20,016 Pero (MI)) containing the following: 10% fetal bovine serum, 10% l-glutamine and 800 μg/ml Geneticin (Gibco).

**Vectors.** PcDNA3.1 KV11.1 A [32] was linearized with *Ssp*I (NEB) and purified (MACHEREY-NAGEL Nucleospin gel and PCR clean up) before electroporation.

pRc/CMV KV1.3 (kindly gifted by Dr. H. Wulff.) was linearized with *Bgl*II (NEB) and purified (MACHEREY-NAGEL Nucleospin gel and PCR clean up) before electroporation.

**CHO cells electroporation.** 107 CHO cell clones have been resuspended in 650 μl of PBS + 45 μg of linearized plasmid in an Electroporation Cuvettes (Biorad 0.2 cm gap). After being incubated on ice for 10 min cells have been electroporated in a Gene Pulser Xcell Total System (Biorad). The magnitude of applied exponential wave pulse was 320 V, 900 μF. Electroporated cells have been immediately placed in ice for 5 min and then resuspended in 12 ml of complete medium in a T75 flask.

After 12 h at 37 °C 5% CO2, the medium was replaced and electroporated CHO cells resuspended in 20 ml of selection medium containing Geneticin (800 μg/ml). Cells have been seeded in two 96-well plates at 37 °C 5% CO2 and single cell clones selected.

Rb^+^ efflux using Ion Channel Reader (ICR) technology. Cells were plated on 96-well plates 24 h before the experimental day and maintained until a ∼90% confluence per well was achieved. On the experimental day, the culturing media was removed from each well and cells were then incubated (37 °C in 5% CO2) with a Rb^+^-loading buffer containing (in mM): 150 NaCl, 2 CaCl2, 0.8 NaH2PO4, 1 MgCl2, 5 glucose, 25 HEPES, and 5.4 RbCl, pH 7.4. After a 3-h incubation, the Rb^+^-loading buffer was replaced with a standard potassium buffer (K-standard buffer) with an identical composition to the Rb-loading buffer but with KCl instead of RbCl. Test compounds were diluted to their final concentration in the K-standard buffer. The 96-well plates were then incubated (37 °C in 5% CO2) for 30 min. To activate the opening of potassium channels, the K-standard solution was removed and cells were stimulated with a depolarizing buffer (Depo-buffer) containing 50 mM KCl (37 °C in 5% CO2) for 10 min. The Depo-buffer also contained test compounds were needed, to avoid drug washout.

After channel activation, supernatants were collected in a separate row of the plate, while cells were lysed 250-μl of 1% PBS-Triton. Before starting each well measurement, a calibration curve was obtained with ICR8000 (Aurora Biomed Inc., Vancouver, BC, Canada) by using standard Rb^+^ concentrations and the R^2^ value was used as a parameter to assess the reliability of the readings. Each reading run consisted of a 100-μl sampling of the supernatant followed by the corresponding lysate for each well. Rb^+^ efflux was used to quantify channel activity and was represented as the ratio of the Rb^+^ content of the supernatant to the total Rb^+^ in each well:

(1) $$Fractional\;Rb^{+}\;efflux=\frac{\;{\left[Rb^{+}\right]}_{sup}}{{\left[Rb^{+}\right]}_{sup}+\;{\left[Rb^{+}\right]}_{lys}}$$

To compare data, each Rb^+^ efflux was then normalized according to the following equation:

(2) $$Normalized\;efflux=\frac{{\left[Rb^{+}\right]}_{frac}-{\left[Rb^{+}\right]}_{basal}}{{\left[Rb^{+}\right]}_{max}-{\left[Rb^{+}\right]}_{basal}}$$

Where [Rb^+^]_frac_ is the fractional efflux from each well (see Eq. (1)), [Rb^+^]_basal_ is the unstimulated fractional efflux, [Rb^+^]_max_ is the maximal fractional efflux. The dose-response curves were fitted using a four parameters logistic regression (4PL) with the following equation:

(3) $$y=\frac{1}{1+10ˆ\left(\left(\log\;{IC}_{50}-x\right)*Hill\;Slope\right)}$$

**Patch-clamp recordings**. On the experiment day, cells were detached mechanically (HEK-293) or enzymatically (CHO) and plated on 35 mm Petri dishes in the culture medium and subsequently kept in an incubator at 37 °C for at least 1 h to promote cell adhesion to the plate. The recorded cells were visually identified at 40× magnification with a Nikon Eclipse TE300 microscope (Nikon Instruments Inc.) equipped with a Photometrics CoolSNAP CF camera (Teledyne Photometrics, Tucson AZ). Before the recording, the culture medium was replaced by the following solution containing (in mM): 130 NaCl, 5 KCl, 2 CaCl2, 2 MgCl2, 10 HEPES, 5 Glucose (E_K_ = −83 mV), pH of 7.4. The internal pipette solution contained (in mM): 140 K+ fluoride, 1 CaCl2, 2 MgCl2, 10 HEPES–NaOH, 11 EGTA, pH 7.2. Membrane currents were recorded at room temperature (∼25 °C) in the whole-cell configuration of the patch-clamp technique, patch pipettes were pulled from borosilicate glass capillary tubes and their resistance was 4–5 MΩ. The pipette capacitance was manually compensated up to 90–95% after the reaching of a stable gigaseal (≥4 GΩ). For experimental protocols and data acquisition, the Multiclamp 700A equipment and pCLAMP 9.2 software (Molecular Devices, Sunnyvale, California, USA) were used. The recordings were low-pass filtered at 5 kHz and digitally sampled at 25 kHz.

Whole-cell outward hERG1 currents were activated by 4-s depolarizing pulses to +20 mV from a holding potential of −80 mV, and tail currents were recorded during 6-s repolarizing steps to −50 mV.

To measure the outward and the inward tail potassium currents we used the following recording protocol: starting from a holding potential (hp) of −80 mV the cells were progressively depolarized with steps ranging from −40 up to +60 mV (step interval 10 mV, 1-s duration) and subsequently repolarized to a hp of −40 mV. The cells were then hyperpolarized to −120 mV for 500 ms to evoke the hERG1 tail-current. To specifically block hERG1 currents, E4031 (1 μM) was applied for 1 min. The net hERG1-mediated currents were then calculated *post-hoc* by subtracting the currents recorded in the presence of E4031 from those obtained under control conditions.

hKv1.3 potassium currents on CHO-hKv1.3 cells were evoked by using a double sweep protocol with an inter-sweep interval of 30s: the first sweep, starting from a hp of 0 mV, was followed by a pulse to +60 mV and used to measure leak current, the second sweep, with a hp of −80 mV, was followed by a pulse to +60 mV and used to measure the potassium current. Net traces (leak-subtracted) were obtained by subtracting the one recorded in the first sweep from the current recorded in the second one. Psora-4 (1 μM, 1 min) the most potent selective Kv1.3 inhibitor [33] was used to test channels’ functionality. Resting membrane potential (V_REST_) values were measured in I-0 mode. The I–V curves were fitted using a Boltzman function with the following equation:

(4) $$y=\frac{1}{1+\exp\left(\frac{V_{50}-V}{k}\right)}$$

**Statistics**. Unless otherwise stated, categorical variables are presented with counts and proportions, while continuous ones are presented as the mean ± standard error of the mean (SEM) or median with IQR (interquartile range). Data statistical analysis was performed using GraphPad Prism v6.0 (GraphPad Software, San Diego, California, USA) and IC_50_ relationships were determined with a four-parameter logistic regression fit normalized to maximum and minimum efflux. Data are given as mean values ± SEM, with n indicating the number of independent experiments. For comparisons between the two groups, we used Student's t-test. All statistical tests were two-tailed with a significance level of 0.05.

## 3 Results

**CHO-hERG1 and CHO-Kv1.3 clone screening**. Our initial experiments were directed to the identification with the ICR8000 system of CHO-transfected cell clones (respectively with hERG1 and hKv1.3 channels) showing a much higher rubidium efflux compared to their wild-type (untransfected) counterpart. These experiments allowed us also to evaluate the basal (unstimulated) rubidium efflux which corresponded to 37.2 ± 1.1% for the CHO-hERG1 cells and 33.0 ± 1.3% for the CHO-Kv1.3 cells.

**Fig. 1:**
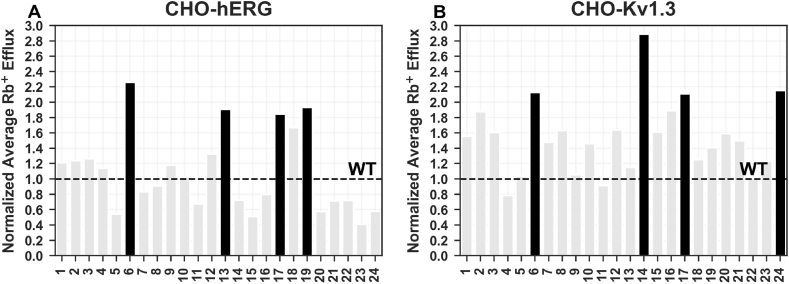
CHO-transfected clones screening. Comparison of the rubidium effluxes between A) the 24 CHO-hERG1 clones and B) the 24 CHO-Kv1.3 clones object of this study. The efflux values are normalized to the efflux measured in the CHO-wt (here shown as a dashed line).

The measurements at ICR8000 system then allowed us to identify, among the different clones tested (24 for CHO-hERG1 and 24 for CHO-hKv1.3 respectively), the 4 best in terms of efflux when compared to the CHO-wt (Fig. 1A–B).

Therefore, as regards the cells transfected with hERG1, the clones named 6, 13, 17 and 19 showed a 2.3-fold, 1.9-fold, 1.8-fold and 1.9-fold increase of the rubidium efflux respectively when compared to the wild-type. On the other hand, among the cells transfected with hKv1.3 the 6, 14, 17 and 24 clones respectively showed a 2.1-fold, 2.9-fold, 2.1-fold and 2.2-fold increase.

**Fig. 2:**
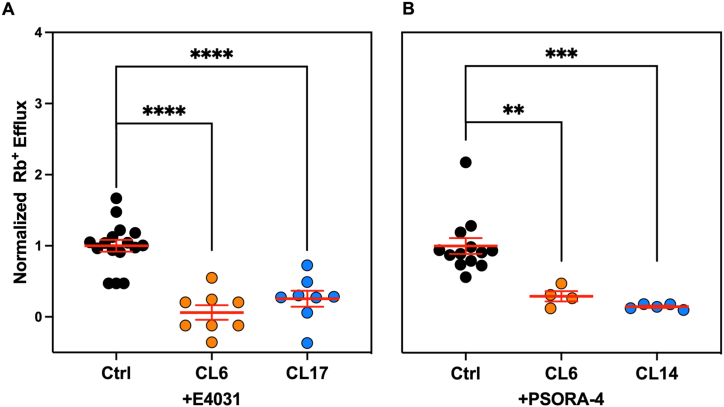
CHO-transfected clones pharmacological test. Bar graph showing the effects of A) E4031 and B) PSORA-4 at the concentration of 10 μM on the two of the best clones among CHO-hERG1 and CHO-Kv1.3 cells performed with the rubidium efflux assay. Values are normalized to the control condition and here reported as a percentage. For statistical significance, two-tail unpaired T-test was used. *p < 0.0332; **p < 0.0021, ***p < 0.0002 and ****p < 0.0001.

**Pharmacology**. So far we wanted to assess the functionality of the two most promising clones of each group. For this purpose, we repeated the rubidium efflux assay under control conditions and in the presence of E4031, a specific blocker for the hERG1 channels, and PSORA-4 for the hKv1.3 channels, both at a concentration of 10 μM. The analysis showed a significant decrease in the rubidium efflux by 94.0% for the hERG1 clone 6 (n = 8, p < 0.0001 unpaired *t*-test) and 74.6% for the hERG1 clone 17 (n = 8, p < 0.0001 unpaired *t*-test) (Fig. 2A). Similarly we observed a significant decrease in the rubidium efflux by 70.6% for the hKv1.3 clone 6 (n = 4, p = 0.004 unpaired *t*-test) and of 85.0% for the clone 14 (n = 5, p = 0.0003 unpaired *t*-test) (Fig. 2B).

**Fig. 3:**
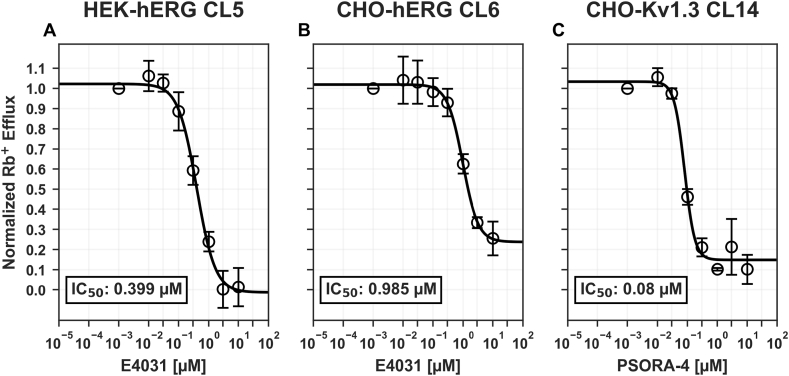
Transfected clones high-throughput dose-response curves. A) E4031 on HEK-hERG1 transfected cells (CL5, IC_50_ = 0.399 μM) and B) on CHO-hERG1 transfected cells (CL6, IC_50_ = 0.985 μM). C) PSORA-4 on CHO-Kv1.3 transfected cells (CL14, IC_50_ = 0.08 μM). Values are normalized to the control condition.

We then continued the functional evaluation in more detail by evaluating the dose-response curves of E4031 and PSORA-4 on the most promising clones as well as on the HEK-hERG1 CL5 cell line (previously developed in our laboratory) as a reference. The application of increasing concentrations allowed us to determine the E4031 IC_50_ which were respectively 399 nM for the HEK-hERG1 cells (Fig. 3A, n = 5) and 985 nM for CHO-hERG1 C6 (Fig. 3B, n = 6). The same assessment with PSORA-4 on CHO-hKv1.3 C^6^ showed and IC_50_ of 80 nM (Fig. 3C, n = 6). All the dose-reponse curves were fitted with equation (1).

**Fig. 4:**
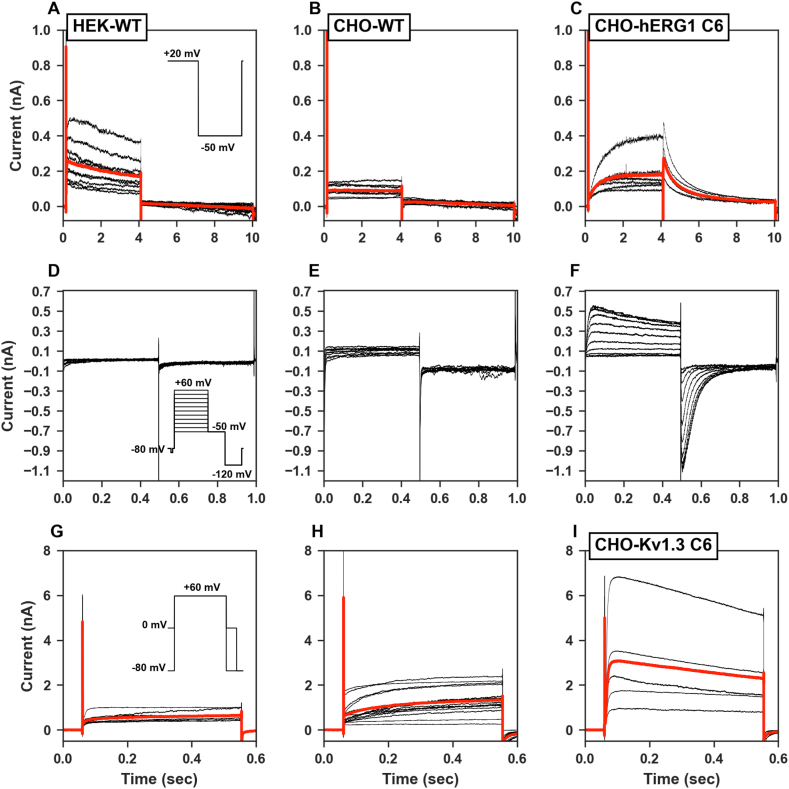
Representative traces of outward potassium currents evoked in A) HEK-wt, B) CHO-wt and C) CHO-hERG1 C6 clones (red: overprinted average traces). The insets represent the pulse protocols used for the electrophysiological recordings. D-F) Representative traces of both outward and inward potassium currents on the same clones. G-I) Representative traces of outward potassium currents evoked in A) HEK-wt, B) CHO-wt and C) CHO-Kv1.3 C6 clones (red: overprinted average traces).

**Patch-clamp electrophysiology**. We started the comparison between the two methods, patch clamp electrophysiology and high-throughput rubidium efflux assay, by recording the potassium outward currents in the two reference clones HEK-wt and CHO-wt and comparing them with the hERG1 overexpressing CHO-hERG1 C6 clone. As expected, we observed a significantly smaller potassium currents in wild-type clones (HEK-wt: 42.0 ± 6.2 pA, p < 0.0001 n = 9; CHO-wt: 46.2 ± 12.3 pA, p = < 0.0001 n = 15) when compared to the CHO-hERG1 C6 (269.0 ± 40.9 pA, n = 9) (Fig. 4A-C). The statistical comparison between the two wild-type clones did not show any significance (p = 0.80). On the other hand, we also observed an inward tail current on CHO-hERG1 C6 cells that were absent on both wild-type clones (Fig. 4D–F). Regarding to the Kv1.3-mediated currents, again as expected, we observed a significantly smaller potassium currents in wild-type clones (HEK-wt: 191.9 ± 52 pA, p = 0.0044 n = 9; CHO-wt: 192.4 ± 50 pA, p = 0.0006 n = 13) when compared to the CHO-Kv1.3 C6 (2732 ± 1005 pA, n = 5) (Fig. 4G–I). The statistical comparison between the two wild-type clones again did not show any significance (p = 0.994).

We then wanted to verify if there was a match between what was observed by the rubidium efflux assay and the data obtained with the patch-clamp method. For this purpose, we also performed whole-cell recordings on CHO-hERG1 and CHO-Kv1.3 clones.

**Fig. 5:**
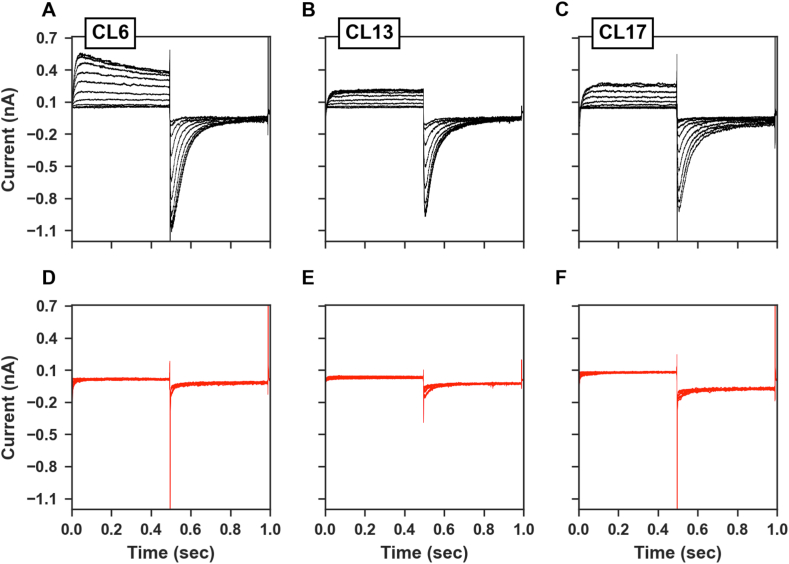
A-C) Representative outward and tail potassium current recordings measured in hERG1-transfected clones (6, 13 and 17) in control conditions. D-F) Representative outward and tail potassium current recordings measured in the same clones (6, 13 and 17) in the presence of 1 μM E4031.

The outward potassium current for the CHO-wt was on average 46.2 ± 12.3 pA (n = 15) and the 4 selected CHO-hERG1 clones all showed an increase in the potassium outward current compared to the CHO-wt respectively by 5.8-fold for clone 6 (268.8 ± 40.9 pA, n = 9), 7-fold for the clone 13 (324.1 ± 126.7 pA, n = 8) and 4.1-fold for the clone 17 (187.6 ± 36.8 pA, n = 5) (Fig. 5A–C). Furthermore, the inward tail potassium current for the CHO-wt was on average 289.8 ± 31.6 pA (n = 15) and for the transfected clones was again increased respectively by 2.7-fold for clone 6 (787.7 ± 93 pA, n = 9), 2.0-fold for the clone 13 (593.3 ± 117.1 pA, n = 8) and 2.2-fold for the clone 17 (646.9 ± 84.6 pA, n = 5) (Fig. 5A–C).

**Fig. 6:**
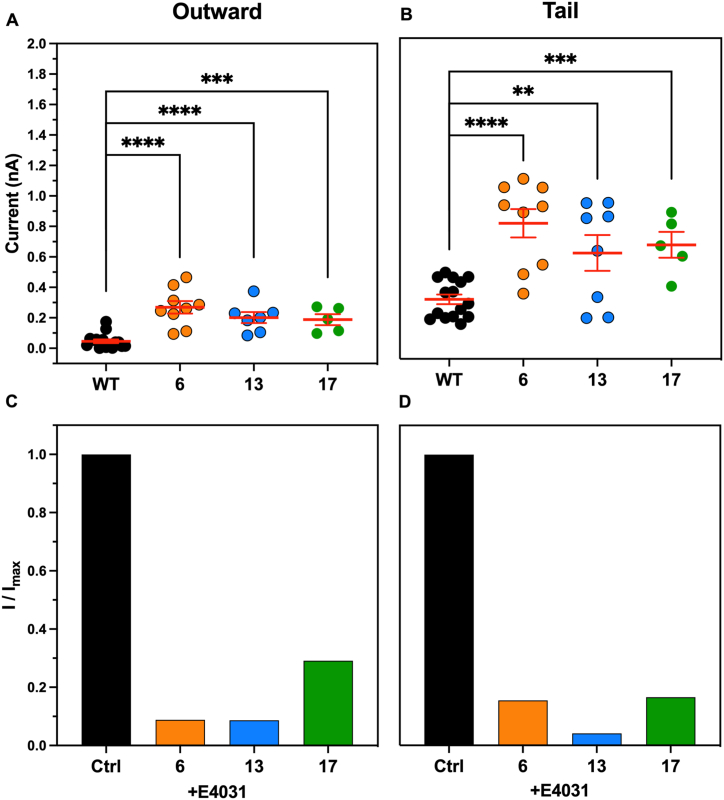
A-B) Average outward and tail potassium currents measured in each clone in control conditions. For statistical significance, two-tail unpaired T-test was used. *p < 0.0332; **p < 0.0021, ***p < 0.0002 and ****p < 0.0001. C-D) Average outward and tail potassium currents measured in each clone in the presence of 1 μM E4031.

The application (1 min) of E4031 at the concentration of 1 μM reduced both the outward currents by 91.7% for the clone 6 (22.2 pA), 93.8% for the clone 13 (20.2 pA) and 57.8% for the clone 17 (79.1 pA) and the inward currents by 80.8% for the clone 6 (151.3 pA), 93.6% for the clone 13 (38 pA) and 78.0% for the clone 17 (142.2 pA) (Fig. 5D–F). Average data of outward and tail potassium currents recorded in control conditions and in the presence of 1 μM E4031 are reported in Fig. 6A-D.

For a more detailed characterization of the clones under examination we measured the I–V curves for both outward and tail hERG1 currents by fitting with the Boltzmann equation (2) described before.

**Fig. 7:**
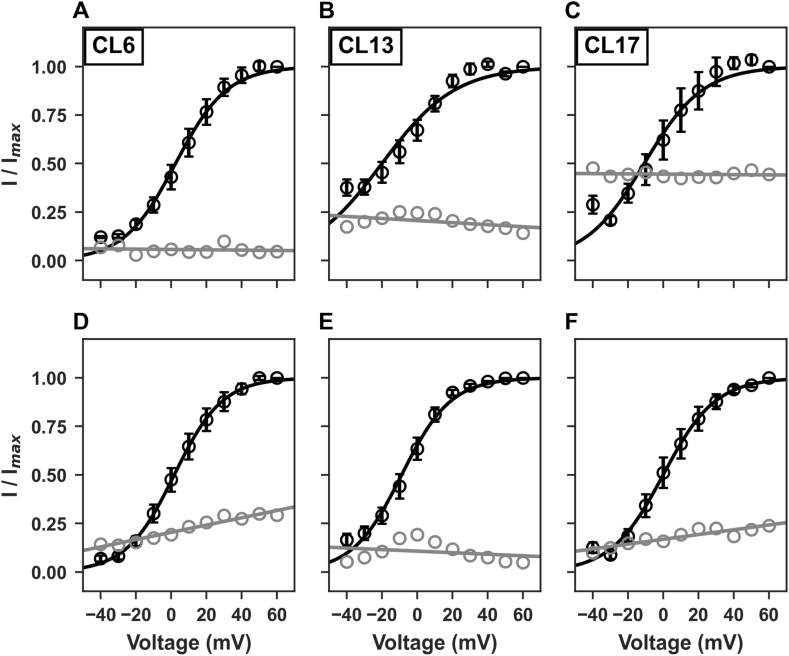
Electrophysiological properties of hERG1 current. A-C) I–V plots of outward hERG1 currents measured in hERG1-transfected clones (respectively 6, 13 and 17) in control conditions (black) and in the presence of 1 μM E4031 (red). D-F) I–V plots of tail hERG1 currents measured in the same clones in control conditions (black) and in the presence of 1 μM E4031 (red).

Outward potassium current amplitudes, normalized to the maximum outward current amplitudes, were used to construct the activation curves shown in Fig. 7A–C (n = 9 cells). The threshold voltage for hERG1 outward currents activation for transfected clones 6, 13 and 17 is close to −40 mV and it is fully activated with voltage steps to +50, +20 and + 40 mV respectively (Fig. 7A–C). When fit as a Boltzmann function, the half-maximum activation voltages (V1/2) for the hERG1 outward currents were 7.8 ± 3.6 mV, −0.3 ± 4.3 mV and 0.4 ± 6.0 mV for clone 6, 13 a 17 respectively.

On the other hand, tail potassium current amplitudes, normalized to the maximum tail current amplitudes, were used to construct the activation curves shown in Fig. 7D–F (n = 9 cells). In this case, the threshold voltage for hERG1 tail currents activation for the same clones is close to −40 mV and it is fully activated with voltage steps to +40, +20 and + 60 mV respectively (Fig. 7D–F). When fit as a Boltzmann function, the half-maximum activation voltages (V1/2) for the hERG1 inward currents were 2.3 ± 3.5 mV, −3.4 ± 2.7 mV and 1.6 ± 4.3 mV for clone 6, 13 and 17 respectively.

**Fig. 8:**
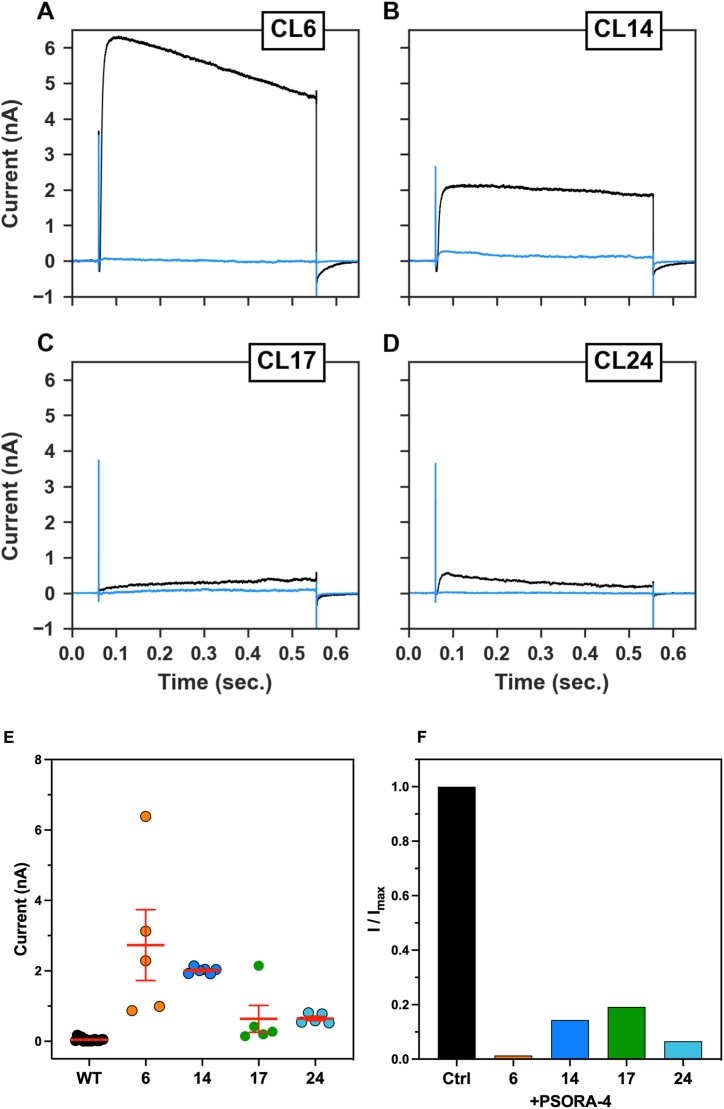
Representative traces of CHO-Kv1.3 clones A) clone 6, B) clone 14, C) clone 17 and D) clone 24 recorded in control conditions (black) and in the presence of 1 μM PSORA-4 (blue). Pulse protocols used are the same shown in Fig. 4G. E) Average outward potassium currents measured in each clone in control conditions and F) in the presence of 1 μM PSORA-4.

We then continued the study with cell clones transfected with Kv1.3. The Kv1.3-transfected clones compared to the wild-type counterpart (average current 46.2 ± 12.3, n = 15) also showed an increase in the potassium outward currents respectively of 59.1-fold for clone 6 (2732 ± 1005 pA, n = 5), 43.6-fold for the clone 14 (2012 ± 34.1 pA, n = 5), 13.8-fold for the clone 17 (636.3 ± 380.5 pA, n = 5) and 14-fold for the clone 24 (646.5 ± 61 pA, n = 5) (Fig. 8A–D). These currents were respectively reduced by 98.7% for the clone 6 (85.3 pA), 85.6% for the clone 14 (289.3 pA), 80.8% for the clone 17 (38.1 pA) and 93.4% for the clone 24 (38.5 pA) after 1 min application of PSORA-4 at a concentration of 1 μM (Fig. 8E-F).

Therefore, the data we obtained with patch-clamp recordings are consistent with those measured by the rubidium efflux assay and confirmed not only the successful overexpression of both hERG1 and Kv1.3 channels, but also the reliability and congruence of the measurements obtained with the high-throughput system.

## 4 Discussion

The aim of this study was to develop a rapid method to evaluate the transfection efficiency of hERG1 and hKv1.3 channels of significant importance, both in physiological and pathological fields, respectively on the HEK-293 and CHO cellular models.

Transfection by electroporation allowed us to overcome the intrinsic genetic instability of CHO cells. This method, compared to lipofectamine based transfection, guarantees a higher rate of integration of the transgenic DNA into the genome of the host cells. It therefore guarantees a greater probability of success in the creation of stable clones of CHO cells.

With this method we generated a total of 48 clones divided respectively into 24 clones transfected with hERG1 (CHO-hERG1) and 24 clones transfected with hKv1.3 (CHO-hKv1.3). We therefore used the ICR8000 high-throughput ion channel reader to evaluate, more quickly than conventional methods, which of these clones showed a higher rubidium efflux. This parameter, being correlated with the transfection efficiency, allowed us to quickly highlight the CHO-hERG1 and CHO-hKv1.3 clones that actually over-expressed the transfected genes coding for the channels object of this study. We therefore chose those clones showing a greater increase in rubidium efflux compared to the non-transfected controls (CHO-wt) using as a threshold value of 1.5-fold for the CHO-hERG1 clones and 2-fold for the CHO-hKv1.3 clones.

So far, we chose the two best clones respectively in the CHO-hERG1 and in the CHO-hKv1.3 group, and we evaluated the response of the transfected channels to their blockers of choice, respectively E4031 for hERG1 and Psora-4 for hKv1.3, again exploiting the rapidity and high yield of the ICR-8000 ion channel reader. As expected, the blockers produced an efflux blockade of ∼70–80% for the hERG1 clones and ∼80–90% for the hKv1.3 clones. Having thus obtained the confirmation that the functionality of the channels had not been in some way altered by the electroporation method, the next step was to characterize more in detail the action of these drugs by evaluating dose-response curves.

As expected, we observed an upward shift in the IC_50_ values when compared to the IC_50_ values found in the literature and obtained with conventional methods. Indeed the IC_50_ of HEK-hERG1 clones measured with the rubidium assay showed a value of 0.4 μM, while the value measured on the same cells with the patch clamp method is reported to be 48 nM [31]. Similarly, we obtained an IC_50_ in CHO-hKv1.3 clones of 80 nM, while the data available in literature report an IC_50_ of 3 nM [33]. The measurements carried out using the classic patch clamp method, unlike the rubidium assay, are indeed performed on a single cell where the drug delivery is directly on the cell. The rubidium assay instead, for each measurement, provides the overall ion efflux contribution of 40.000–50.000 cells and this would explain the shift of the IC_50_, albeit congruous, that we observed beeing the patch clamp experiments performed with a fast perfusion system.

To continue the characterization of the transfected cell clones we therefore conducted experiments using the patch-clamp technique in whole-cell configuration. These measurements allowed us to further validate the transfection efficacy through a method that represents the golden standard, although more limited in terms of execution speed and yield when compared to the ICR8000 high-throughput ion channel reader, confirming the observations previously obtained.

As expected, patch-clamp recordings on clones previously screened with the rubidium assay showed a 2- to 7-fold increase in outward and inward potassium currents when compared with those measured in the wild-type counterparts. Subsequent pharmacological tests, carried out using the specific blockers E4031 and Psora-4 on patch-clamp experiments, once again demonstrated the non-compromised functionality of the ion channels under examination.

Therefore we can conclude that the ICR8000 high-throughput ion channel reader represents a reliable tool when applied to a rapid and high-throughput screening of multiple cell clones, as well as to the pharmacological-functional study of ion channels on a large scale. Moreover, since this system does not require physical contact with the membranes in order to measure the ion flow, it is not affected by the problems relative to obtaining a stable gigaseal, avoiding patch break and trapping the cells which are instead implicated in other methods (e.g. patch clamp, IonFlux™). Indeed, considering the congruence in the shift of the IC_50_ values, a conversion is easily obtainable which allows to relate the pharmacological data obtained with the rubidium assay with those obtained through conventional methods, confirming once again the validity of this assay in the pharmacological field. Further applications could, in example, be addressed to the systematic study of mutations affecting potassium and other ion channels, as well as to the screening of newly large scale synthesized molecules.

## Author contribution statement

Alberto Montalbano; Cesare Sala; Ginevra Chioccioli Altadonna: Conceived and designed the experiments; Performed the experiments; Analyzed and interpreted the data; Wrote the paper. Andrea Becchetti: Conceived and designed the experiments; Analyzed and interpreted the data; Wrote the paper. Annarosa Arcangeli: Conceived and designed the experiments; Analyzed and interpreted the data; Contributed reagents, materials, analysis tools or data; Wrote the paper.

## Funding statement

This research was funded by the University of Florence (to A.A.). This work was supported by 10.13039/501100005010Associazione Italiana per la Ricerca sul Cancro (AIRC, grant no. 1662, 15627) to A.A.; PRIN Italian Ministry of University and Research (10.13039/501100003407MIUR) 20174TB8KW to A.A..

## Data availability statement

Data will be made available on request.

## Additional information

No additional information is available for this paper.

## Declaration of competing interest

The authors declare the following financial interests/personal relationships which may be considered as potential competing interests: Annarosa Arcangeli reports financial support was provided by Italian Association for Cancer Research.
